# A leprosy clinical severity scale for erythema nodosum leprosum: An international, multicentre validation study of the ENLIST ENL Severity Scale

**DOI:** 10.1371/journal.pntd.0005716

**Published:** 2017-07-03

**Authors:** Stephen L. Walker, Anna M. Sales, C. Ruth Butlin, Mahesh Shah, Armi Maghanoy, Saba M. Lambert, Joydeepa Darlong, Benjamin Jewel Rozario, Vivek V. Pai, Marivic Balagon, Shimelis N. Doni, Deanna A. Hagge, José A. C. Nery, Kapil D. Neupane, Suwash Baral, Biliom A. Sangma, Digafe T. Alembo, Abeba M. Yetaye, Belaynesh A. Hassan, Mohammed B. Shelemo, Peter G. Nicholls, Diana N. J. Lockwood, on behalf of the Erythema Nodosum Leprosum International STudy Group

**Affiliations:** 1 Department of Clinical Research, Faculty of Infectious and Tropical Diseases, London School of Hygiene and Tropical Medicine, London, United Kingdom; 2 Leprosy Laboratory, Oswaldo Cruz Institute, Fiocruz, Rio de Janeiro, Brazil; 3 Department of Medicine, The Leprosy Mission International, Dhaka, Bangladesh; 4 Department of Dermatology and Mycobacterial Research Laboratories, The Leprosy Mission Nepal, Anandaban Hospital, Kathmandu, Nepal; 5 Department of Dermatology, Leonard Wood Memorial Center for TB and Leprosy Research, Cebu, Philippines; 6 Department of Family Medicine, Suisse Clinic, Addis Ababa, Ethiopia; 7 Department of Medicine, The Leprosy Mission Hospital, Purulia, India; 8 Bombay Leprosy Project, Mumbai, India; 9 Department of Dermatology, ALERT Center, Addis Ababa, Ethiopia; Oxford University Clinical Research Unit, VIET NAM

## Abstract

**Objectives:**

We wished to validate our recently devised 16-item ENLIST ENL Severity Scale, a clinical tool for measuring the severity of the serious leprosy associated complication of erythema nodosum leprosum (ENL). We also wished to assess the responsiveness of the ENLIST ENL Severity Scale in detecting clinical change in patients with ENL.

**Methods:**

Participants, recruited from seven centres in six leprosy endemic countries, were assessed using the ENLIST ENL Severity Scale by two researchers, one of whom categorised the severity of ENL. At a subsequent visit a further assessment using the scale was made and both participant and physician rated the change in ENL using the subjective categories of “Much better”, “somewhat better”, “somewhat worse” and “much worse” compared with “No change” or “about the same”.

**Results:**

447 participants were assessed with the ENLIST ENL Severity Scale. The Cronbach alpha of the scale and each item was calculated to determine the internal consistency of the scale. The ENLIST ENL Severity Scale had good internal consistency and this improved following removal of six items to give a Cronbach’s alpha of 0.77. The cut off between mild ENL and more severe disease was 9 determined using ROC curves. The minimal important difference of the scale was determined to be 5 using both participant and physician ratings of change.

**Conclusions:**

The 10-item ENLIST ENL Severity Scale is the first valid, reliable and responsive measure of ENL severity and improves our ability to assess and compare patients and their treatments in this severe and difficult to manage complication of leprosy.

The ENLIST ENL Severity Scale will assist physicians in the monitoring and treatment of patients with ENL. The ENLIST ENL Severity Scale is easy to apply and will be useful as an outcome measure in treatment studies and enable the standardisation of other clinical and laboratory ENL research.

## Author summary

Erythema nodosum leprosum (ENL) is a severe, painful complication of leprosy, which can occur before, during or after successful treatment of the infection. ENL is characterised by severe pain and the development of new painful skin lesions. Other organ systems are often affected. ENL may continue to affect people for many years leading to disability, significant loss of income and sometimes death. ENL often requires prolonged treatment with corticosteroids, thalidomide or other drugs that modulate the immune system. Thalidomide is the most effective treatment but is not widely available or affordable in many leprosy endemic settings. This results in many people taking high doses of corticosteroids for prolonged periods and being at risk of severe adverse effects. In order to assess the efficacy of different treatments for ENL it is important to be able to compare individuals before and after treatment. This is difficult to do in a complex, multi-system disorder such as ENL. To overcome this problem we have developed a scale, which is easy to use, to measure the severity of ENL. In this article, we describe the validation of this scale, which ensures that it is a useful measure of ENL severity.

## Introduction

Erythema nodosum leprosum (ENL) is a severe inflammatory complication of borderline lepromatous (BL) leprosy and lepromatous leprosy (LL). ENL affects up to 50% of individuals with LL and 5–10% of BL leprosy patients [1, 2]. A bacterial index of four or more is also a risk factor for developing ENL. ENL may occur before, during or after successful completion of anti-mycobacterial multi-drug therapy (MDT)[2].

ENL causes inflammation in many systems and is characterised by severe pain, tender cutaneous skin lesions, fever, joint and bone pain, iritis, orchitis, lymphadenopathy and neuritis [3]. Most patients have multiple episodes of painful inflammation extending over several years [2, 3].

ENL is associated with a deleterious impact on health related quality of life (HRQoL)[4], increased mortality[5] and severe economic hardship for affected individuals and their families[6].

The Erythema Nodosum Leprosum International STudy (ENLIST) Group[7] aims to improve the understanding of the mechanisms which cause ENL, improve the evidence to guide treatment decisions of individuals with ENL and improve access to effective treatments. The ENLIST Group includes clinicians and laboratory scientists with extensive experience in the treatment and investigation of the causes of ENL based at institutions in eight countries.

The cause of ENL is unclear. It is associated with a complex array of immune activation and consequent inflammation, which requires immunosuppression. ENL skin lesions may show features of vasculitis and there is evidence of neutrophil and lymphocyte activation. The role of immune complexes in ENL remains unproven. Patients are treated with corticosteroids, clofazimine and thalidomide either alone or in combination, and less commonly other immunomodulatory agents, which are used for prolonged periods of many months or years [3]. Many patients require high doses of corticosteroids to control their disease and this leads to complications and deaths associated with long-term use of these drugs[8]. Thalidomide is usually effective but is not available in many countries or it is severely restricted because of the risk of teratogenicity. Other adverse effects occur with thalidomide and these have been reported to occur in 25–68% of individuals [9–12]. The neuropathy caused by thalidomide during its use to treat other conditions is approximately 20% but there are no good data for the frequency of thalidomide-induced neuropathy in patients with ENL[13]. The identification of other agents for controlling ENL has been identified as a priority for patients in countries where thalidomide is prohibited or highly restricted, unaffordable, ineffective or poorly tolerated [14].

The evidence for choosing the appropriate treatment for ENL is limited. There have been eight small, randomised treatment studies of ENL since the introduction of MDT [9–12, 15–17]. Only 269 patients were enrolled into these studies and just three studies with a total of 53 participants reported allocation concealment and blinding [15, 16]. Determining outcome measures for clinical studies in complex, multisystem disorders such as ENL is difficult. Quantitative severity scoring systems provide one possible outcome measure.There have been several scoring systems employed in studies of ENL however none have been validated [9, 18–21]. Unpublished (non-validated) scales have been shown to be a source of bias in randomised controlled trials [22, 23]. A Cochrane review highlighted the difficulty in comparing treatment studies in ENL and recommended the development of validated severity scales[14].

We developed a 16 item scale, the ENLIST ENL Severity Scale (EESS), for measuring the severity of ENL[24]. We applied and critically appraised three previously published scales for ENL that had not been validated and used regression analysis of data from our cross-sectional study of the clinical features of ENL[3] to enable us to develop the EESS.

The scale incorporates assessments of pain and wellbeing using visual analogue scales (VAS), fever, skin signs, oedema, orchitis, ocular inflammation, joint and bone involvement, nerve assessments and urinalysis. We wished to validate the EESS and determine the minimal important difference (MID). MID is a concept used to determine whether an outcome is clinically relevant and relates to the smallest difference in score which is perceived as beneficial[25].

## Methods

Participants who gave written, informed consent were recruited from seven centres: DBLM Hospital, Nilphamari, The Leprosy Mission Bangladesh; Oswaldo Cruz Institute, Rio de Janeiro, Brazil; the ALERT Center, Addis Ababa, Ethiopia; Bombay Leprosy Project, Mumbai, India; The Leprosy Mission Hospital, Purulia, India; Anandaban Hospital, The Leprosy Mission Nepal and the Cebu Skin Clinic, the Leonard Wood Memorial Center, Cebu, Philippines.

Ethical approval was obtained from the Ethics Committee of the London School of Hygiene and Tropical Medicine (Reference 10370), The Leprosy Mission International Bangladesh Institutional Research Board, Brazilian National Ethical Review Board (CAAE: 12834613.4.0000.5248), AHRI-ALERT Ethical Review Committee (PO12/16), Ethics Committee of the Managing Committee of the Bombay Leprosy Project (BLP/OO/TRC/76A/2015), The Leprosy Mission Trust India Ethics Committee, the Nepal Health and Research Council (106/2011), Institutional Ethical Committee/Institutional Ethical Regulatory Board of Cebu Leprosy and Tuberculosis Research Foundation, Inc. (CLTRFI/LWM-IEC 2015–005).

Individuals were eligible to participate in the study if they met any of the following criteria:

- Diagnosed with BL leprosy or LL within 24 months of enrolment (and who did not have ENL or a history of ENL)
- History of ENL with no evidence of active ENL and not requiring treatment for ENL
- Diagnosed with or receiving treatment for ENL

For the purposes of the study ENL was defined as a patient with leprosy who had crops of tender cutaneous or subcutaneous lesions or was on treatment for ENL (whether it was active or not).

The type of ENL was categorised as acute, recurrent or chronic which were defined as follows:

- **acute** for a single episode lasting less than 24 weeks,
- **recurrent** if a patient experienced a second or subsequent episode of ENL occurring 28 days or more after stopping treatment for ENL
- **chronic** if occurring for 24 weeks or more during which a patient has required ENL treatment either continuously or where any treatment free period had been 27 days or less[5].

Individuals who did not wish to give consent or were diagnosed with leprosy Type 1 reactions were excluded.

Each participant was examined independently by a health worker (usually a doctor but sometimes a physiotherapist and in one centre an experienced leprosy research scientist) who had been trained to use the EESS and by an experienced leprologist who also applied the scale and categorised the ENL as “inactive” or “mild” or “moderate” or “severe”. We did not attempt to standardise the categorisation of ENL by the experienced leprologists. Neither assessor (nor the participant) was aware of the result of the other assessor’s examination. The time interval between the two assessments was kept as short as practicable.

At a subsequent visit, the MID of the EESS was determined by applying the scale to individuals after treatment and asking both the participant and the examining leprologist to independently categorise the change as: “much better”, “somewhat better”, “no change” (or “about the same” for physicians), “somewhat worse” or “much worse”. The leprologist had performed one of the original assessments at the first visit but did not apply the EESS on the second occasion and was blinded to the result (as was the participant). The EESS, on this occasion, was applied by the same health worker as at the initial visit whenever possible. The MID methodology was only used for participants who had been categorised as having mild or moderate or severe ENL at the first set of assessments.

All data including demographic, clinical and EESS were collected on data collection forms specifically designed for the study. The anonymised data were entered into a password protected Access database at each centre and subsequently merged. The data were analysed using Stata 14 (StataCorp. 2015 *Stata Statistical Software*: *Release 14*. College Station, TX: StataCorp LP).

### Statistical methods

We wished to recruit 300 participants as this would provide more than 10 study subjects per scale item [26].

The internal consistency or reliability was assessed using Cronbach’s alpha. An alpha between 0.7 and 0.9 is considered acceptable[27]. The contribution of each item in the scale was assessed by calculating Cronbach’s alpha for the scale if that item were removed.

The ability of the scale to discriminate between patients with different clinical severity categories was determined using analysis of variance. The threshold for accepting statistical significance was p<0.05.

Inter-observer reliability was evaluated using Intra-Class Correlation of the total score of each examiner using a two-way analysis of variation (5% level of significance) and the strength of agreement criteria of Landis and Koch[28]. A Bland Altman plot of the difference between pairs of observations and the mean of those pairs was used to highlight any potential systematic differences between assessors

Receiver operator characteristic curves were used to determine cut off points for mild, moderate and severe reactions.

The ability of the scale to reflect the change in ENL was calculated as the mean of the change in severity associated with each of the reported outcomes “Much better”, “somewhat better”, “somewhat worse” and “much worse” compared with “No change” or “about the same” (for physician rated change).

## Results

447 individuals were recruited between 13^th^ May 2015 and 16^th^ July 2016. 336 (75.3%) were male and 110 (24.7%) female. 19 physicians classified the severity of the 210 participants with active ENL. The demographic and clinical features are summarised in Table 1.

**Table 1:** Characteristics of the participants in the validation study.

			n (%)
**Gender**	**Male**		337 (75.4)
	**Female**		110 (24.6)
**Age in years (mean±SD)**			36.2±14.0
**Categories of participants**	**BL/LL without ENL**		146 (32.7)
	**History of ENL**		77 (2.9)
	**Inactive ENL**		13 (17.2)
	**Mild ENL**		66 (14.8)
	**Moderate ENL**		106 (23.7)
	**Severe ENL**		39 (8.7)
**Type of ENL (n = 210)**	**Acute**		60 (28.6)
	**Recurrent**		38 (18.1)
	**Chronic**		98 (46.7)
	**Not specified**		14 (6.7)
**MDT status**	**Naive**	**All**	26 (5.8)
		**ENL**	4
	**Current**	**All**	267 (59.9)
		**ENL**	102
	**Completed**	**All**	153 (34.3)
		**ENL**	104
**Treatment for ENL at time of enrolment**	**No treatment**		96 (45.7)
	**Treatment**		114 (54.3)

54.3% of the 210 individuals with ENL were receiving treatment for their ENL at the time of enrolment. Of the nine drug regimes used, prednisolone (57.2%), prednisolone and clofazimine (24.6%), thalidomide (6.5%), thalidomide and prednisolone (4.3%) and clofazimine alone (2.9%) were the most common.

### Scale testing

Initially only 14 of the 16 items were considered for inclusion in the severity scale. The VAS Wellbeing item was excluded because we wished to maintain a strictly clinical focus. Orchitis was also omitted as we wished to try and produce a gender-neutral scale. The items showing the lowest levels of correlation were inflammation of the eyes due to ENL, urinalysis and the items related to sensory and motor nerve function.

The internal consistency of the 14 potential items for inclusion in the scale was assessed using Cronbach's alpha producing an initial value of 0.7413. The series of analyses reported were based on the data from those individuals who had been classified as having mild or moderate or severe ENL (n = 210). Removing eye inflammation due to ENL and urinalysis increased the value of alpha to 0.7633. Removing the count of nerves with sensory NFI due to ENL and the count of nerves with motor NFI due to ENL further increased alpha to 0.7672. Removal of any further items brought a reduction in alpha, confirming the inclusion of the remaining 10 items in the scale.

The derived 10 item scale was analysed separately for men to see if the addition of orchitis significantly altered the internal consistency. Using the data for men alone the 10 item scale has a Cronbach alpha of 0.7633 which increases to 0.7645 with the addition of orchitis. The increase in alpha did not result in greater internal consistency for men compared to men and women combined.

### Unidimensionality and discrimination of the 10 item scale

Principal component analysis showed a general factor to which all 10 items contributed accounting for 33.4% of the total variance. A second “pain” factor contributed 16.0% of the total variance. It contrasted VAS pain, bone pain, inflammation of joints and nerve tenderness with items describing the number, extent and inflammation of skin lesions and lymphadenopathy.

**Fig 1:**
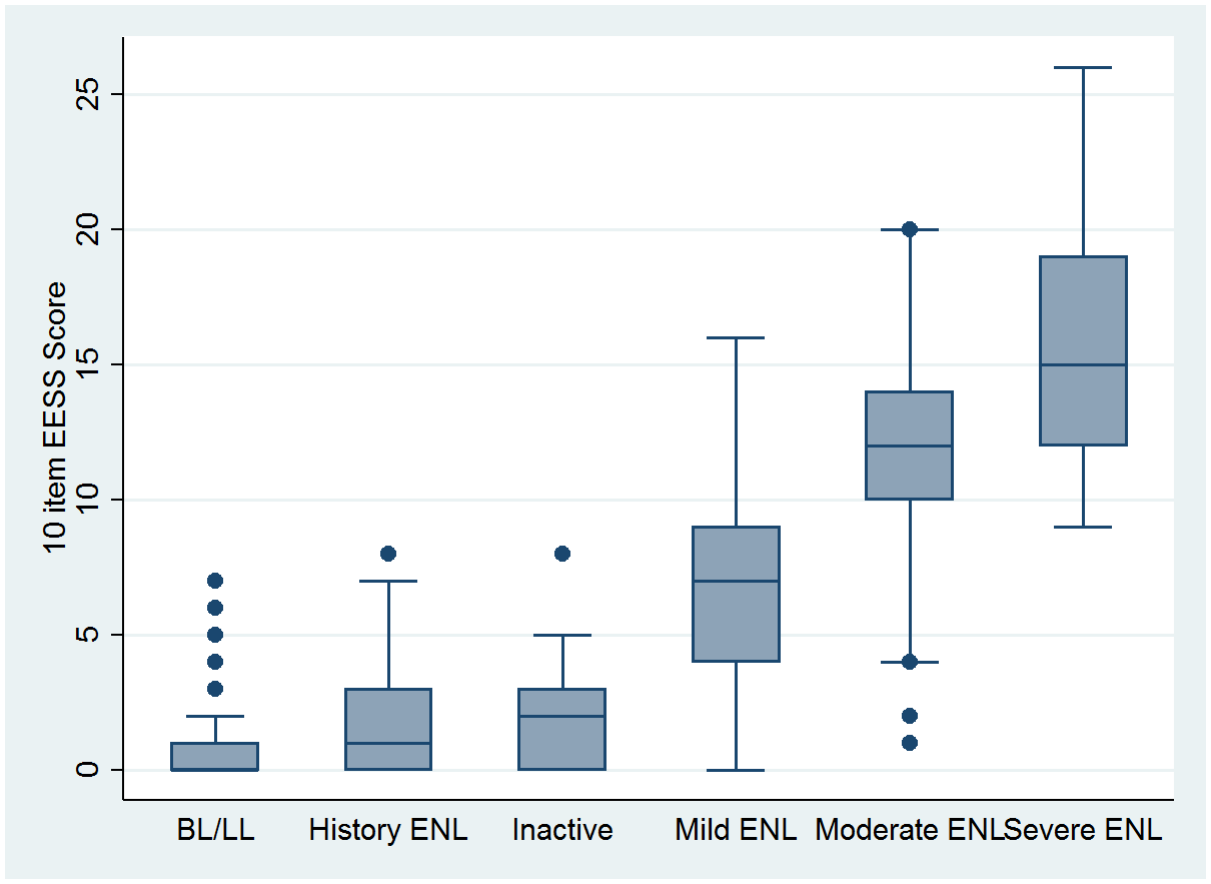
Box plot of the 10 item scale by expert classification showing medians, interquartile ranges and minimum and maximum scores.

The 10 item scale discriminated well between patients with active ENL and those without. Fig 1 shows the distribution

The difference in scores between the non-ENL group and those categorised as having mild ENL were significant (p<0.001, parametric and non-parametric test).

A threshold value differentiating between those who were classified as having “moderate” ENL and those with “severe” ENL was not identified by either score or ROC curve. However, the difference in mean severity scores was statistically significant (p<0.001, parametric and non-parametric test).

The EESS scores of participants with acute or recurrent or chronic ENL were not significantly different.

The intra-class correlation coefficient assuming random effect for both patients and assessors and individual assessors is 0.797 (95% CI 0.742, 0.843). The strength of agreement is good[28].

**Fig 2:**
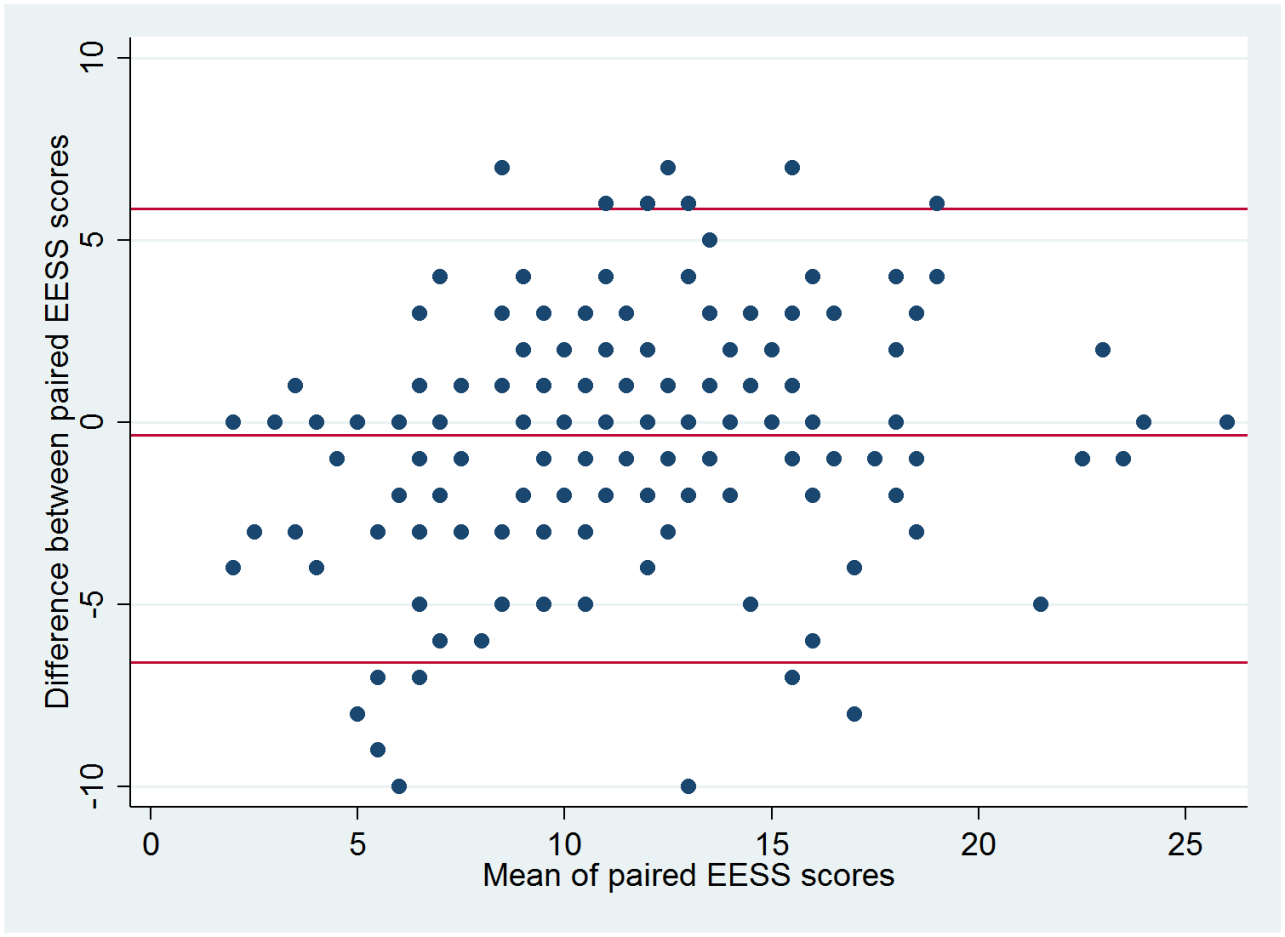
Bland-Altman plot of the two scores derived from the 10 item EESS at the first assessment.

A Bland-Altman plot (Fig 2) showed good agreement between the two assessors of each patient with no evidence of a systematic difference in terms of larger differences for higher severity scores. 15 (7.4%) of the 204 paired assessments fell outside the confidence limits but these were evenly distributed between positive and negative differences.

**Fig 3:**
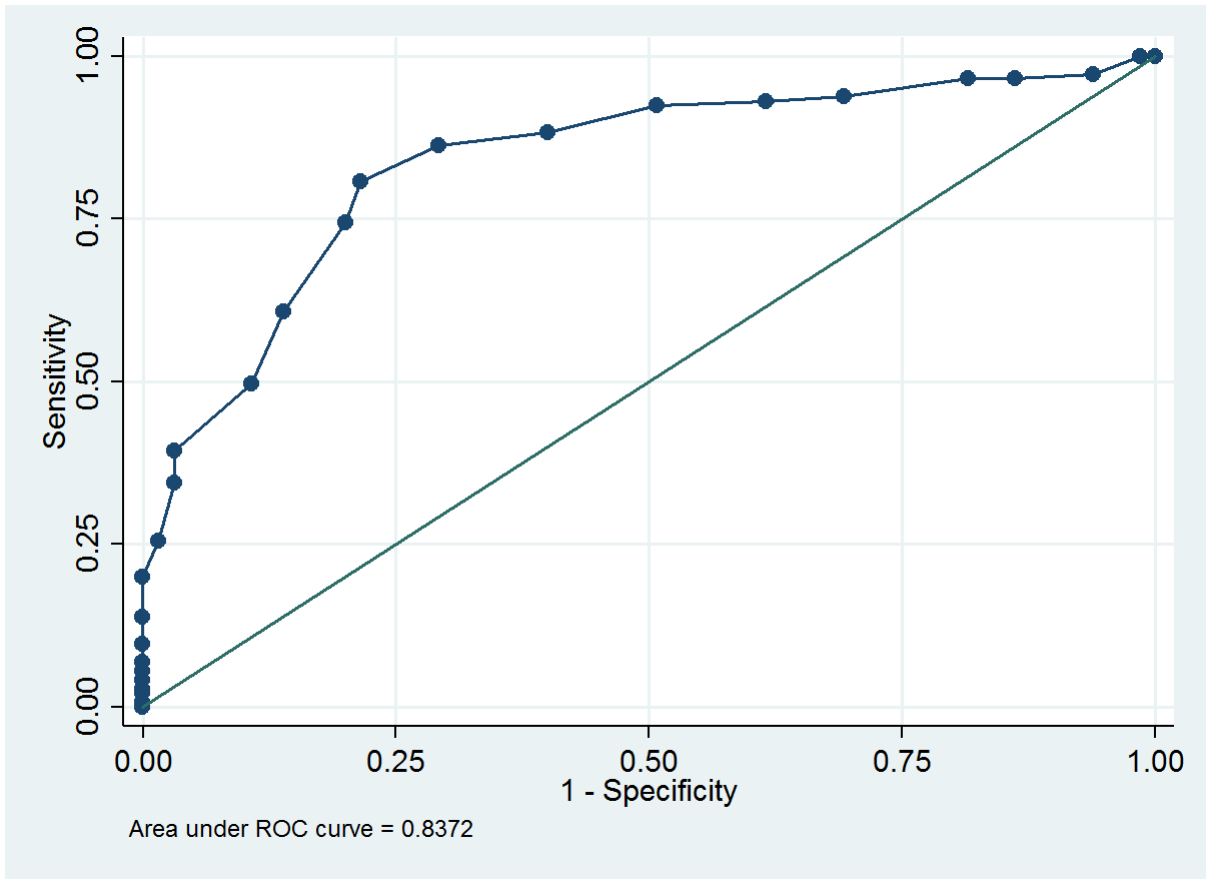
ROC curve for those with mild ENL and those with either moderate or severe ENL.

To determine the cut off score between “mild” and more severe categories of ENL the ROC curve was plotted for patients classified as having “mild” ENL and those with either moderate or severe ENL (Fig 3).

**Fig 4:**
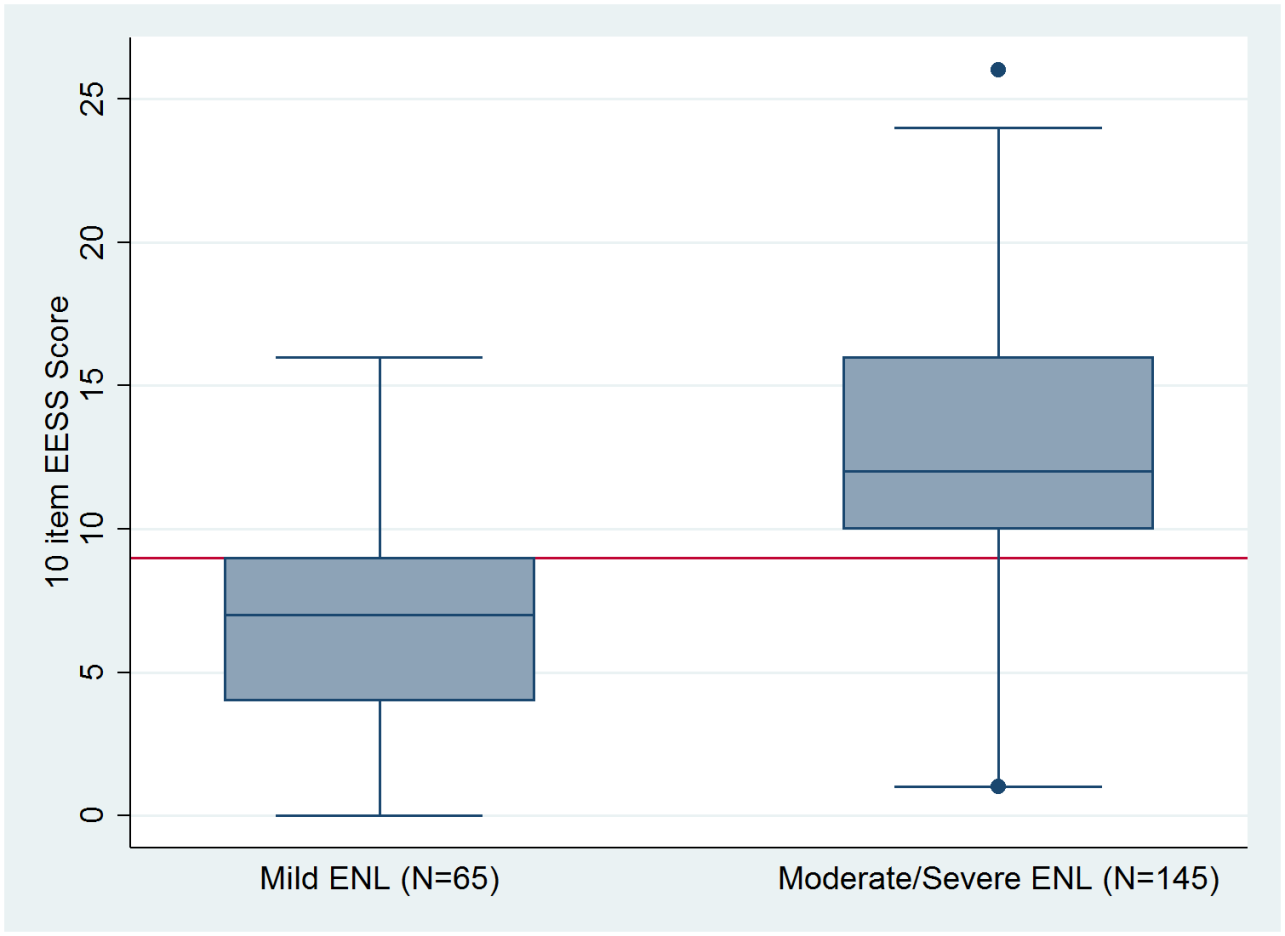
Box plot of 10 item EESS scores for mild compare with moderate and severe ENL. The line in red indicates the cut off between mild ENL and more severe disease.

Mild ENL was determined to be an EESS score of 8 or less and more severe forms scoring 9 or more (Fig 4). The area under the curve for mild and combined moderate and severe ENL is 0.8372. This value indicates that the final scale is a good discriminator between the mild and more severe categories of ENL traditionally used by clinicians.

**Fig 5:**
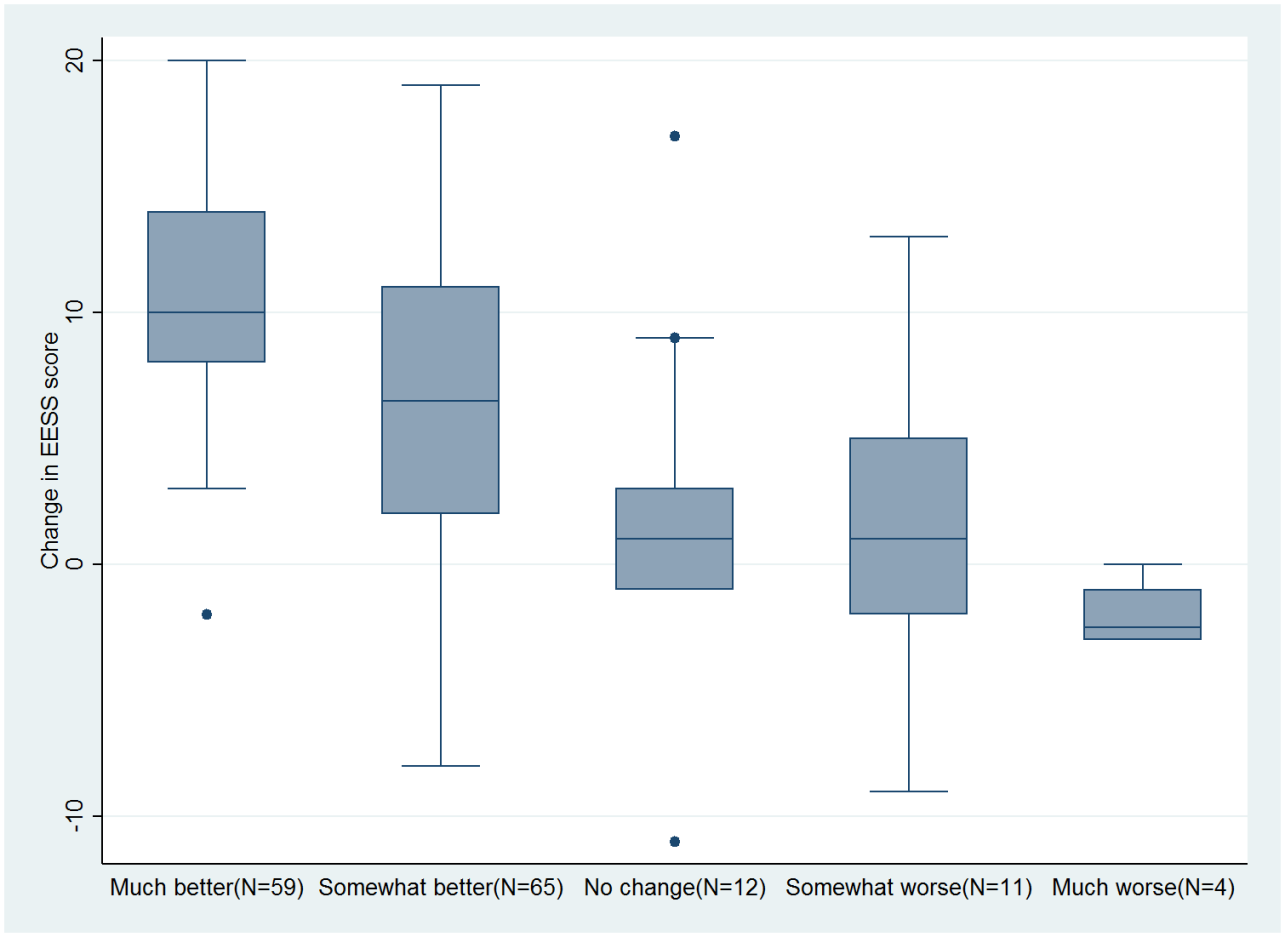
Change in EESS score and participant rated improvement.

**Fig 6:**
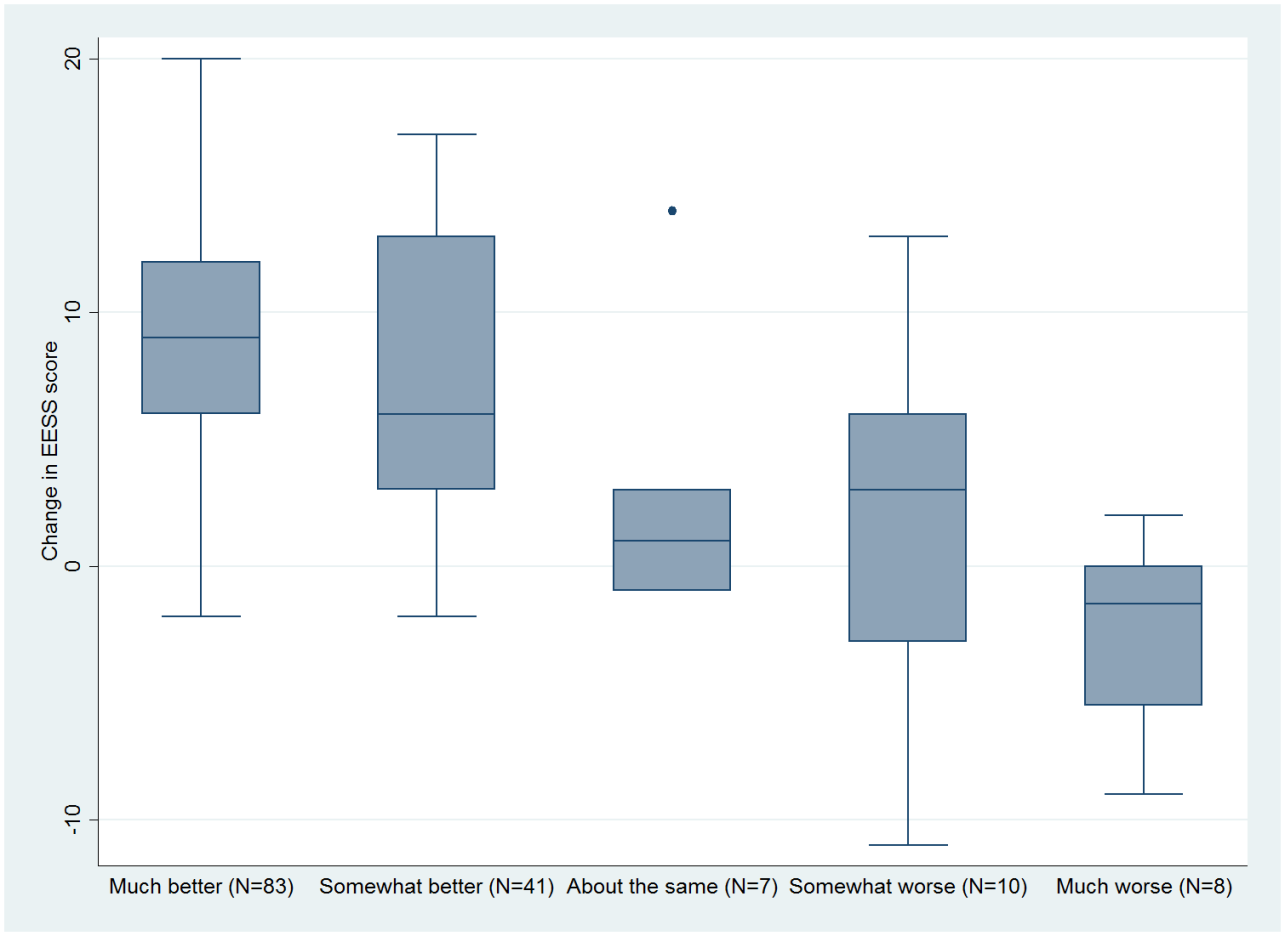
Change in EESS score and physician rated improvement.

152 participants with ENL completed the two sets of assessments. The median interval between these two sets of assessments was 28 days, range 0 to 185.The changes in EESS scores and the participant rated and physician rated improvement are shown in Figs 5 and 6 respectively.

Table 2 shows the mean change in scores for each participant-rated or physician-rated change and the difference from “no change” or “about the same” respectively. The MID was 4.9 for both participants and physicians which equates to a change in EESS score of 5. The change in mean EESS score from baseline to “much better” which resulted in an “important difference” was 8.4 for participants and 7.0 for physicians. This equates to a change in EESS score of 9.

**Table 2:** Change in EESS score and participant and physician rated change with difference from “No change” or “About the same”.

	Participant rated/ Physician rated change				
	Much better	Somewhat better	No change/About the same	Somewhat worse	Much worse
**Mean change in EESS score** **±SD by participant**	10.2 ± 4.4	6.7± 5.7	1.8 ± 6.6	1.3± 6.0	-2.0 ± 1.4
**Difference from “No change”** **(95% CI)**	8.4 (7.3, 9.5)	4.9 (3.8, 6.0)	----------	-0.6 (-2.1, 1.0)	-3.8 (-5.1, -2.6)
**Mean change in EESS score** **±SD by physician**	9.2 ±5.1	7.1 ± 5.7	2.7± 5.3	2.0 ±7.2	-2.6± 3.9
**Difference from “About the same”** **(95% CI)**	7.0 (5.9, 8.1)	4.9 (3.8, 6.0)	-----------	-0.2 (-2.0, 1.6)	-4.8 (-6.2, -3.4)

## Discussion

Leprosy reactions present a major challenge to the successful management of the disease. The adapted version of the EESS is a valid and reliable measure of the severity of ENL. We have been able to show that it discriminates between patients with mild ENL and more severe disease. The scale and accompanying guide (S1 Appendix) are easy for clinicians to use.

A clinical tool to measure the severity of leprosy Type 1 reactions was designed and validated by some members of the ENLIST Group [29]. Type 1 reactions are a major cause of nerve damage in leprosy. The Type 1 reaction severity scale was first validated in Brazil and Bangladesh and in a subsequent study from Ethiopia[30]. It has been shown to reflect change in severity following treatment and has been used in clinical trials of corticosteroids, azathioprine and ciclosporin [31–34]. We believe that the EESS has the potential to be equally important in ENL.

The EESS is gender-neutral following removal of the orchitis item which did not materially affect the internal consistency of the scale. The two items relating to NFI were removed from the final version of the EESS but clinicians should remain vigilant to new NFI occurring in the context of ENL, which we have previously shown to have a prevalence of 22.9% in individuals with ENL[3]. Nerve tenderness is a component of the scale but nerve tenderness does not always accompany NFI. Eye involvement due to ENL is not included in the scale and clinicians will need to be cognizant of this uncommon feature when using the EESS.

We were able to demonstrate that the EESS discriminates between moderate and severe ENL but could not determine a cut off score for the two categories. We did not attempt to standardise physician assessment of the severity of ENL which is a limitation of the study. The overlap of scores in the group categorised as having “moderate” ENL and those with “severe” ENL is likely to have occurred due to variation in physician perception of the construct of ENL severity. The multisystem nature of ENL may mean that different physicians attach different weight to different symptoms or signs when categorising ENL severity. Variation between physician global assessments has also been reported to occur during comparison of different methods for the assessment of flares in systemic lupus erythematosus [35].

The MID of the EESS was determined using both participant and physician reported change. There are no agreed criteria for determining which group should be used for determining MID. It has been argued that physicians are the best judges of change in measures of disease activity or damage, whereas in functional or HRQoL measures it is the affected individual [36]. We felt it was important to assess the responsiveness of the EESS using both groups. The results were concordant with an MID of 5 for both groups. The greatest discordance, of two scale units, between the ratings of participants and physicians occurred for the change of “much better”.

The EESS is the first validated, published scale of ENL severity and is responsive to change in ENL. We plan to use the EESS in future double-blind randomised controlled treatment studies of ENL and believe it will be an important tool for other clinical researchers. The scale will also be useful in providing a standardised way of describing the severity of ENL in patients who participate in immunological and genetic studies. It is equally important that the EESS be incorporated into routine clinical practice where we believe it will help physicians to assess, monitor and treat patients.

## Supporting information

S1 Appendix ENLIST ENL Severity Scale. Validated ENLIST ENL Severity Scale and User Guide.
(PDF)
