# Supplementary material for: A leprosy clinical severity scale for erythema nodosum leprosum: An international, multicentre validation study of the ENLIST ENL Severity Scale
Source: PLoS Negl Trop Dis. 2017 Jul 3;11(7):e0005716. doi: 10.1371/journal.pntd.0005716 (PMC5510881; doi:10.1371/journal.pntd.0005716)
Supplement: S1 Appendix — Validated ENLIST ENL Severity Scale and User Guide. (PDF) [file pntd.0005716.s001.pdf]

S1 Appendix

ENLIST ENL Severity Scale – FINAL VERSION, which supersedes the previously published version[24]

Pain Rating - Visual Analogue Scale (Ensure line is 100 mm long)

How severe is your pain today? Mark the line below with an **X** to indicate how bad you feel your pain is ***today***

____

No Pain **Worst possible Pain**

ITEM		SCORES				
		0	1	2	3	**SCORE**
**1**	**VAS – Pain (mm)**	0	1-39	40-69	70-100	
**2**	**Fever** (in **°C**)	None (37.5 or less)	No fever now but history of fever in last 7 days	37.6-38.5	38.6 or higher	
**3**	**Number of ENL skin lesions**	None	1-10	11-20	21 or more	
**4**	**Inflammation of ENL skin lesions**	Non tender	Redness	Painful	Complex	
**5**	**Extent of ENL skin lesions**	0	1-2 regions	3-4 regions	5-7 regions	
**6**	**Peripheral oedema**	None	1 site of Hands or Feet or Face	2 sites	All three sites (Hands and Feet and Face)	
**7**	**Bone pain**	None	Present on examination but does **not** limit activity	Sleep or activity disturbed	Incapacitating	
**8**	**Inflammation of Joints and/or digits due to ENL**	None	Present on examination but does **not** limit activity	Sleep or activity disturbed	Incapacitating	
**9**	**Lymphadenopathy due to ENL**	None	Enlarged	Pain or tenderness in 1 group	Pain or tenderness in 2 or more groups	
**10**	**Nerve tenderness due to ENL**	None	Absent if attention distracted	Present even if attention distracted	Patient withdraws limb on examination	
	**TOTAL**					

User Guide for the ENLIST ENL Severity Scale - FINAL VERSION which supersedes previously published version

The score for each item should be **added** together to obtain the ENLIST ENL Severity Scale score.

Mild ENL is categorised as an ENLIST ENL Severity Scale score of **8 or less.**

The **Minimal Important Difference** of the ENLIST ENL Severity Scale is **5.**

SCALE ITEM	NOTES
**1. VAS Pain**	Instruct the patient to point to the position on the line to indicate how much pain they are ***currently*** feeling. The far left end indicates 'No pain' and the far right end indicates 'Worst possible pain'.<br>Take the measurement (in **mm**) using a ruler from the **LEFT** end of the line to the centre of the cross.<br>***Ensure that the line when reproduced from this document is 100 mm long.***
**2. Fever**	Take temperature (in **°C**) using a thermometer.<br>If the temperature is GREATER than 37.5°C the patient has a fever. If it is less than or equal to 37.5°C the patient scores 0 for this item UNLESS they give a history of having had a fever in the last 7 days in which case they score 1. The cause of the fever does not need to be established.
**3. Number of ENL skin lesions**	***Note: only skin lesions due to ENL are to be considered for this item.***
**4. Inflammation of ENL skin lesions**	***Note: only skin lesions due to ENL are to be considered for this item.***<br>The term complex refers to the following type of skin lesions: vesicular, bullous, pustular, erythema multiforme-like, panniculitis, necrotic, ulcerated.<br>***If the participant fulfils criteria for more than one score then the highest scoring criteria should be used.***<br>For example if there are red ENL skin lesions and some are ulcerated or vesicular or pustular then the patient scores 3 because "complex" lesions are present.
**5. Extent of ENL skin lesions**	***Note: only skin lesions due to ENL are to be considered for this item.***<br>The separate regions are:<br>a) Head and neck<br>b) Left upper limb<br>c) Right upper limb<br>d) Torso –front (including genitals)<br>e) Torso back (including buttocks)<br>f) Left lower limb<br>g) Right lower limb
**6. Peripheral oedema due to ENL**	The three sites to be considered are the face, hands and feet.<br>*Both feet count as one site. Both hands count as one site.* Oedema thought to be due to treatment such as corticosteroids or thalidomide should not be counted.

**7. Bone pain**	Bone pain is distinct from pain or tenderness of the joints. It is most usually elicited by palpation of the subcutaneous border of the tibia.
**8. Inflammation of Joints and/or digits due to ENL**	***Note: only joint inflammation due to ENL is to be considered for this item.***<br>Inflammation of the joint will be present if there is any of the following: pain or tenderness, redness, swelling or heat. It them must be determined if any of these are sufficiently severe to meet the criteria of the scores. If more than one joint is affected the most severely affected joint is used to determine the score.
**9. Lymphadenopathy due to ENL**	The lymph node groups to be examined are:<br>a) Head and neck (including the supraclavicular fossae)<br>b) Axillary<br>c) Inguinal<br>***Note: Lymph node groups on the different sides of the body are separate for example: left axillary and right axillary. Therefore there are 6 lymph node groups for the purposes of the scale.***
**10. Nerve tenderness due to ENL**	Any peripheral or cutaneous nerve tenderness due to ENL is to be considered. If the participant fulfils criteria for more than one nerve then the highest scoring nerve should be used.<br>***The most severely affected nerve should be used.***<br>***Where the examiner suspects that neuropathic pain is being elicited then this should be disregarded.***

Definitions of "complex" skin lesions

Bulla is defined as a visible accumulation of fluid within or beneath the epidermis more than 0.5cm

Erythema multiforme-like lesions are atypical ENL lesions resembling those of erythema multiforme and include macular, papular or urticarial lesions, as well as the classical iris or 'target lesions'.

Panniculitis inflammation of the subcutaneous adipose tissue

Pustule an accumulation of free pus

Target lesions are defined as less than 3 cm in diameter and have three or more zones, usually a central area of dusky erythema or purpura, a middle paler zone of oedema and an outer ring of erythema with a well-defined edge

Ulceration a break in the epithelial surface (the epidermis in the skin)

Vesicle is defined as a visible accumulation of fluid within or beneath the epidermis 0.5cm or less in diameter
